# Improving the prediction of protein binding sites by combining heterogeneous data and Voronoi diagrams

**DOI:** 10.1186/1471-2105-12-352

**Published:** 2011-08-23

**Authors:** Joan Segura, Pamela F Jones, Narcis Fernandez-Fuentes

**Affiliations:** 1 Leeds Institute of Molecular Medicine, Section of Experimental Therapeutics. University of Leeds. Leeds, LS9 7TF, UK; 2 Leeds Institute of Molecular Medicine, Section of Molecular Gastroenterology. University of Leeds. Leeds, LS9 7TF, UK

## Abstract

**Background:**

Protein binding site prediction by computational means can yield valuable information that complements and guides experimental approaches to determine the structure of protein complexes. Predictions become even more relevant and timely given the current resolution of protein interaction maps, where there is a very large and still expanding gap between the available information on: (i) which proteins interact and (ii) how proteins interact. Proteins interact through exposed residues that present differential physicochemical properties, and these can be exploited to identify protein interfaces.

**Results:**

Here we present VORFFIP, a novel method for protein binding site prediction. The method makes use of broad set of heterogeneous data and defined of residue environment, by means of Voronoi Diagrams that are integrated by a two-steps Random Forest ensemble classifier. Four sets of residue features (structural, energy terms, sequence conservation, and crystallographic B-factors) used in different combinations together with three definitions of residue environment (Voronoi Diagrams, sequence sliding window, and Euclidian distance) have been analyzed in order to maximize the performance of the method.

**Conclusions:**

The integration of different forms information such as structural features, energy term, evolutionary conservation and crystallographic B-factors, improves the performance of binding site prediction. Including the information of neighbouring residues also improves the prediction of protein interfaces. Among the different approaches that can be used to define the environment of exposed residues, Voronoi Diagrams provide the most accurate description. Finally, VORFFIP compares favourably to other methods reported in the recent literature.

## Background

The experimental characterization of the structure of protein complexes by X-ray crystallography, Nuclear Magnetic Resonance (NMR) or Electron Microscopy (EM) cannot keep pace with the ever-expanding volume of interactome data. Moreover, weak or transient interactions are very difficult to crystallize, NMR has clear limitations with regard to the size of the protein complexes that are tractable, and EM often does not provide adequate resolution. Computational tools, such as protein binding site predictions and protein docking, offer alternatives to describe protein interactions by providing theoretical structural models of protein complexes (e.g. [1]). Indeed, computational and experimental methodologies are complementary rather than mutually exclusive; for example protein binding site predictions can guide mutational analyses aimed at charting protein interfaces.

Residues located at protein interfaces present distinct physicochemical properties. Hydrophobic residues predominate in permanent complexes, although charged residues often form part of interfaces [2-5]. Interface residues also have both higher solvent accessibilities [2,6] and lower crystallographic B-factors [7] than those seen in exposed residues not involved in protein interfaces. Other studies have shown that interface residues are evolutionarily conserved [8-10] although this has been questioned in several reports [11,12]. Finally, interface residues are less prone to sample alternative side-chain rotamers to minimize entropic cost upon complex formation [13-15].

The features described above can be used individually or in combination to predict protein interfaces (see [16] for a recent review). Methods used to predict protein binding sites include those based on patch analysis [17] and those based on Neural Networks, including methods developed by Fariselli et al. [18], Ofran and Rost [19], and Porollo and Meller [20]. The latter includes the neighbourhood or environment of residues as input data, defining the environment as the residues enclosed in a Euclidean distance threshold, which results in more accurate predictions. Neuvirth et al [21] proposed a method that utilizes secondary structure, hydrophobicity and experimental B-factors among other structural features. A support vector machine integrating six structural and chemical features was proposed by Bradford et al. [22] and later refined using Bayesian Networks [23]. A parametric score function based on sequence conservation and structural information has been also proposed [24]. More recently, Sikić et al [25] proposed a Random Forest ensemble classifier to predict interface residues using a 9-residue sliding window that includes sequence and structural information.

Despite these existing methods, the accurate generic prediction of protein interfaces is not resolved. The lack of a clear understanding of protein-protein interaction hinders the development of more accurate methods, and thus new approaches and ideas are needed. Also, as new experimental data emerge, new prediction algorithms can be devised that outperform their predecessors, thus providing better tools for the scientific community. Here, we describe a novel, structure-based, computational method: Voronoi Random Forest Feedback Interface Predictor (VORFFIP). VORFFIP is a two-steps Random Forest (RF) ensemble classifier that integrates a set of input variables accounting for structural features, energetic terms, evolutionary conservation, and crystallographic B-factors. In addition, VORFFIP uses Voronoi Diagrams (VDs) to define the local environment of exposed residues; this provides a more accurate description of the effect of the neighbourhood than can be provided by either the sliding window or Euclidean distance approaches. VDs have been used in a number of applications to study atomic packing of proteins [26], define protein interfaces [27], identify residue-residue contacts [28], define pockets in proteins [29] or define molecular surfaces [30]. However, VDs have not been used in the context of protein binding site predictions to define the environment of exposed residues.

The performance of VORFFIP has been comprehensively assessed using different combinations of input data and environment definitions, identifying the combinations that lead to the best performance. Finally, VORFFIP outperformed other prediction methods under similar benchmarking conditions.

## Results and Discussion

The aim of this work was to identify which features, environment descriptors and their combinations yielded the best results when predicting protein binding sites. This would highlight the best approach to distinguish exposed residues that are likely to be part of a protein interface from those that are not. To that end, a comprehensive study using VORFFIP was performed evaluating the results against widely used performance indicators. Unless specifically noted, all the results were obtained in a 5-fold cross validation test using the B100 dataset. The B100 dataset was derived from Benchmark 3.0 [31] after discarding antigen-antibody complexes (see additional file 1, Material and Methods section for more information). Protein complexes in the B100 dataset have two representatives: the bound and unbound conformations. The bound conformation was only used to define interfaces (i.e. defining the residues located at protein interfaces); however, training and predictions were performed on the unbound conformations. In this manner, it was ensured that no information from the bound conformation was used during the training and prediction.

### One step vs. two-steps RF

**Figure 1:**
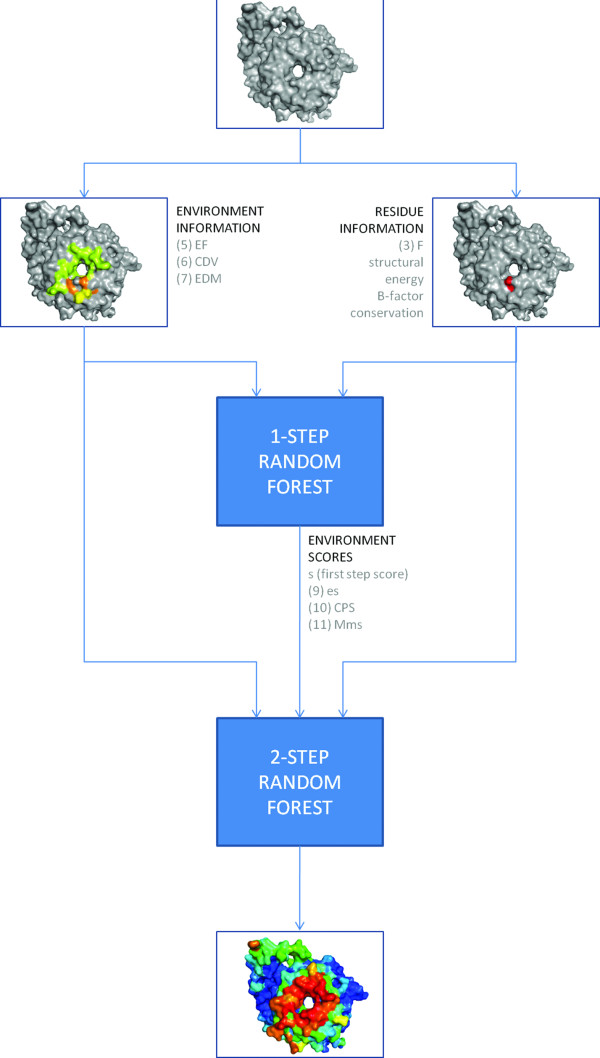
**Overview of VORFFIP method**. The first-step RF uses residue and environment-based features as input variables. The second-step RF also included variables derived from the score values assigned by the first-step RF yielding a final prediction score. Numbers between parentheses refer to the different equations described in the Method section.

As described in the introduction, VORFFIP is a prediction method that relies on a two-step RF ensemble classifier (Figure 1). The difference between the first-step and second-step RF is the use of scores assigned by the first-step RF and the environmental score-derived metrics: *es_i _*(9), CSV (10) and Mms (11) (see Methods section). The logic behind using the second-step RF relates to the observation that residues belonging to the same interface tend to form contiguous patches on the surface. Thus, it would be unlikely that residues with high scores would be mainly neighbours to low scoring residues unless located in the boundary of the interface. It would be expected that the second-step RF would harmonize outliers and generate homogenous and high scores for interface residues by using the first-step RF scores together with the quantification of scores of neighbouring residues (i.e. environmental scores) and thus resulting in better predictions.

Results show that the performance of VORFFIP is improved when the second-step RF is included. The ROC curve obtained on the second-step RF showed higher sensitivity for any false-positive rates (Additional file 1, Figure S1) and the difference in AUC values was statistically significant (*p*-value < 0.01). Both ROC curves were derived using structure, energy, conservation and B-factors together with VD to account for the neighbourhood. However, the same behaviour was observed when using individual sets or combination of features such as structure and conservation and other environment descriptors such as sliding window (data not shown). In terms of precision (P), recall (R), F1-scores and Matthews correlation coefficient (MCC), the second-step RF also produced better results: first-step RF vs. second-step RF; R: 0.50 vs. 0.56; P: 0.36 vs. 0.45; MCC: 0.34 vs. 0.42; F1-scores 0.41 vs. 0.49. Thus, second-step RF and score-derived metrics such as *es_i _*(9) corrected false positives and identified missing hits, thus improving the performance of VORFFIP. Unless otherwise noted, the two-step RF was selected as default predictor.

### Improving predictive power by combining heterogeneous data and using Voronoi Diagrams

A total of 60 combinations of features (structure, energy, conservation and crystallographic B-factors) and environment definitions (VDs, sliding window as in Sikić et al. [25], Euclidian distances as in Porollo and Meller [20] and no-environment) were explored. The predictive performance of single features and 11 combinations are presented in Table 1. The general trend shows that combining features resulted in a statistically significant increase of AUC values. Individual features all performed at a similar level, with B-factors being the poorer predictor in terms of AUC. However, the best performance was achieved when all features were combined and VDs were used as environment descriptor. Different combinations of features yielded different results: no clear improvements were observed when structural information was combined with energy information (*p*-value 0.06; Additional file 1, Table S1) or when B-factor data were added to structure, energy, and conservation (*p*-value 0.58; Additional file 1, Table S1). Finally, evolutionary data (e.g. sequence conservation) did improve predictions in terms of AUC.

**Table 1:** AUC values for different combinations of features and environment definitions

Features	Voronoi Diagrams	Sphere	Sliding Window	Single (no environment)
**s**	0.79	0.75	0.77	0.72
**e**	0.77	0.72	0.75	0.71
**c**	0.76	0.74	0.72	0.65
**b**	0.74	0.71	0.69	0.61
**s+e**	0.78	0.75	0.77	0.73
**s+c**	0.82	0.78	0.81	0.77
**s+b**	0.79	0.75	0.77	0.73
**e+c**	0.81	0.75	0.77	0.76
**e+b**	0.77	0.73	0.75	0.72
**c+b**	0.76	0.74	0.72	0.68
**s+e+c**	0.82	0.78	0.81	0.77
**s+e+b**	0.79	0.75	0.77	0.73
**s+c+b**	0.82	0.78	0.8	0.78
**e+c+b**	0.81	0.75	0.77	0.76
**s+e+c+b**	0.85	0.78	0.81	0.77

The test consisted of a 5-fold cross validation using dataset B100 where interface residues were defined using DIMPLOT (Wallace, et al., 1995). The first column indicates the combination of features used: structural (s), energy (e), conservation (c), and B-factors (b). The second, third, fourth, fifth columns contain AUC values for Voronoi Diagram, sphere (15 Å cut-off), 9-residue sliding window, and single residue (no environment), respectively.

**Figure 2:**
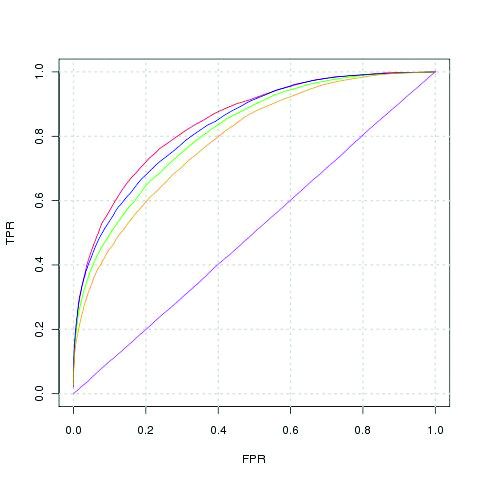
**ROC curves combining structure, energy, conservation and B-factors information and different environment definitions**. Red, green, blue and yellow lines represent ROC curves using VDs, sphere, sliding window, and single residues (i.e. no environment) as environment descriptors respectively. Purple line represents a random prediction.

When evaluating the effect of environment descriptors, in general VORFFIP achieved the best performance when VDs were used. As shown in Figure 2, VDs and the combinations of structural, energy, conservation and B-factors achieved the best performance in terms of AUC values with a higher true positive rate, regardless of the false positive rate when compared to sliding window, sphere or no use of environment information. The difference in AUC between VDs and other environment descriptors was statistically significant (Additional file 1, Table S2).

A similar trend that a combination of all features and VDs gave the best scores was observed with other performance indicators such as MCC, R, P and F1-scores (Additional file 1, Table S3). MCC scores are of special interest due to the ratio between positive and negative cases: the number of exposed residues that do not belong to an interface is much higher than those that do. Both MCC and F1-scores improved when all the sources of information were combined and VD was used to account for the environment, thus resulting in better and more balanced predictions. For the sake of completeness, different Euclidian distance cut-offs between 5 to 20 Å (5 Å binning) were tested. The optimal performance is achieved between 10 and 15 Å cut-off agreeing with a previous observation [20]. However, VORFFIP's predictions were still more accurate when VDs were used to account for the local environment (Additional file 1, Table S4).

### Effect of the environment descriptors

**Figure 3:**
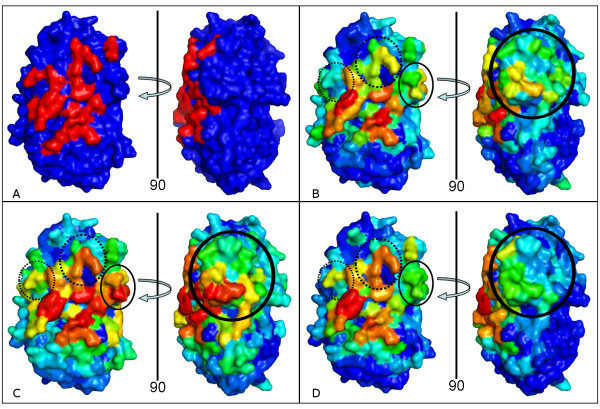
**Evaluating the effect of environment descriptors**. The binding site of CI-2-SUBTILISIN NOVO (PDB code: 2sni, chain E; surface representation) was predicted using structural, energy, conservation, and B-factor information and three different types of environments definitions. (A) Interface as in the crystal structure (highlighted in red). (B) Prediction using a 9-residues sliding window. (C) Prediction using distance threshold (15 Angstroms cut-off). (D) Prediction using VDs. The gradient colour represents score values (s) where: blue (0 ≤ s < 0.5), green (0.5 ≤ s ≤ 0.7), yellow (0.7 ≤ s < 0.9), and red (s > 0.9). Solid and dashed circles represent differences in the prediction of non-interface and interface residues, respectively.

As shown above, the inclusion of environment descriptors had a positive effect on the performance of VORFFIP. In general, any prediction that included environment information was superior that those that did not (Additional file 1, Table S1 and Table S3). However, VDs were superior when compared to sliding windows and spheres due to the combination of a lower rate of false positive and a higher rate of true positive cases. A specific case of this effect is depicted in Figure 3. In general, when VORFFIP uses environment information derived from sliding window, sphere and VDs, high scores are assigned to the main interface patch. However, using information derived from both sphere and sliding window resulted in either low scores assigned to residues in the interface patch or high scores assigned to residues that are not (Figure 3B-C), whereas VDs (Figure 3D) yielded a more accurate and balanced prediction, thus resulting in a sharper and more accurate charting of the protein binding site. It is worth noting that while VDs and sphere descriptors only considered exposed residues, the sliding window approach, which is sensitive to the structural position of the central residue of the window, can include buried residues which might have a negative effect on the performance of the prediction.

### Comparing VORFFIP with previous studies

The algorithm was compared against three recently published methods: SPPIDER [20], WHISCY [24] and the method developed by Sikić et al. [25]. In each case, VORFFIP was trained and tested following the same procedure described in the previous studies and using the same datasets. Also, the definition of interface residues was the same as described in the original publications (Additional file 1, Datasets section for more information).

SPPIDER implements a Neural Network and includes several structural and sequence features as well as information about neighbourhood or environment, considering a residue to be part of the neighbourhood if located within a 15 Å radius sphere centred on the residue of interest. For training and testing, Porollo and Meller [20] derived two non-redundant and independent sets referred to here as the S435 and S149 datasets; VORFFIP was trained using the S435 set and tested using the S149 dataset. The comparison between SPPIDER and VORFFIP (Table 2) shows that VORFFIP achieved higher scores for each of the metrics used to evaluate predictive performance: MCC, Q2, R, P and AUC values (ROC curves are presented in additional file 1, Figure S2).

**Table 2:** Comparing SPPIDER and VORFFIP

METHOD	MCC^a^	Q2(%)^b^	R(%)^c^	P(%)^d^	AUC^e^
VORFFIP	0.58	83.8	74.7	63.4	0.90
SPPIDER	0.42	74.2	60.3	63.7	0.76

(a) Matthew correlation coefficient, (b) Second quartile, (c) Recall, (d) Precision, (e) Area under the ROC curve. VORFFIP values were obtained using the default predictor. SPPIDER values taken from [20].

WHISCY [24] relies on sequence conservation and was benchmarked using the W025 dataset. The SO72 dataset, generated from O333 by removal of any protein complexes whose SCOP [32] superfamily is represented in dataset W025, was used to train VORFFIP. This ensured that no evolutionary relationship, however remote, existed between the training set SO72 and the testing set W025. Comparing to WHISCY, VORFFIP performed better in terms of R, P and MCC scores as shown in Table 3. Individual predictions for each individual protein complex in W025 dataset are also shown in additional file 1, Table S5.

**Table 3:** Comparing WHISCY, WHISCYMATE and VORFFIP

METHOD	R(%)^a^	P(%)^b^	MCC^c^
VORFFIP	47	42	0.38
WHISCY	27	39	0.27
WHISCYMATE	28	36	0.26

(a) Recall, (b) Precision, (c) Matthew correlation coefficient. VORFFIP values were obtained using the default predictor. WHISCY and WHISCYMATE values taken from [24]

Finally, Sikić's method [25] relies on a 9-residue sliding window that includes sequence, secondary structure and several structural features as input variables to a RF classifier. Sikić's method was benchmarked using the O333 dataset on a 3-fold cross validation test. Additional file 1, Figure S3 shows a precision versus recall plot, similar to the one reported in the original publication [25]. As shown, VORFFIP achieved a higher precision at any recall rate (except for first-step RF at recall rates lower than 0.3).

## Conclusions

In this work we present VORFFIP, a novel computational tool for the prediction of protein binding sites. Several studies of protein complexes with known crystal structures have shown that residues at interfaces present unique properties (see Introduction). These properties, which provide information that is specific to structural features, energy terms, evolutionary conservation and crystallographic B-factors of individual residues, have predictive power. However, combining this range of individual features by means of a RF ensemble classifier clearly improved prediction; the combination of information is more powerful than the individual pieces of information. Moreover, the second-step RF further enhanced the performance of the method. The results show that all statistical measures used to gauge the performance of the method showed improvement from the first-step to the second-step RF, and thus incorporating the score values obtained by the first-step RF led to better predictions, probably because of the nature of protein binding sites formed from the contiguous surface patches.

Accounting for the environment of residues also enhanced the accuracy of the prediction. Although this observation is not new, the use of VDs in the framework of protein binding site prediction is novel. VDs not only provide a better approach to define protein interfaces (as shown by Cazals et al [27]) but also sharper and more accurate definition of the local environment of exposed residues as shown by the results presented here. VORFFIP and VDs delivered the best predictions in comparison to other approaches to define the local environment of residues, such as Euclidean distances (spheres) or sliding window. Moreover, there are clear advantages in using VDs, including no requirement for cut-offs (distances or window) and given its nature, it is easy to implement a weighting system based on the number of contacts (see Methods section). Thus, VDs offer a more natural and rational approach for defining the structural environment of residues.

Significant differences were observed between the precision and recall values in the SPPIDER and WHISCY tests. While SPPIDER was trained and tested using a set of protein complexes, i.e. proteins in bound conformation, WHISCY used protein complexes from Benchmark set version 1.0 [33] and version 2.0 [34]. Benchmark sets have two representations for each protein complexes, unbound and bound; predictions were performed only on the unbound version to ensure no bound information was used during prediction. It was found that crystallographic B-factors were very good predictors on the SPPIDER dataset whereas their performance seriously decreased when using the WHISCY dataset. This observation highlights the need for reliable datasets, such as the Benchmark series [31], to properly and fairly benchmark computational methods.

In summary, this paper describes a new computational tool for the prediction of binding sites. VORFFIP is a two-step RF ensemble classifier that relies on a set of input variables that accounts for several aspects of residue and environment-based information. VORFIPP compared favourably against other reported methods. VORFFIP is accessible at http://www.bioinsilico.org/VORFFIP.

## Methods

### Dataset and definition of protein interfaces

Five datasets of protein complexes, termed O333, S435, S149, W025 and B100, were used for benchmarking (B100) and to compare with previous methods (O333, S435, S149, W025). In the case of O333, S435, S149, W025 datasets, different definitions of protein interfaces were used depending on the description in the original publication. Full details are given in additional file 1, Material and Methods section. Briefly, the O333 set corresponds to that compiled by Ofran and Rost [35] and used by Sikic et al. [25]. S435 and S149 correspond to the two sets derived by Porollo and Meller that were used to train and test SPPIDER [20]. The dataset W025 corresponds to both Benchmark 1.0 [33] and 2.0 [34] sets and was used to benchmark WHISCY [24]. Dataset B100 corresponds to Benchmark 3.0 [31] after discarding antigen antibody complexes and was used as an independent set to benchmark VORFFIP under different conditions such as input data and environment definitions. Datasets can be downloaded from http://www.bioinsilico.org/VORFFIP/datasets.html.

### Defining the environment of exposed residues: Voronoi Diagrams (VDs)

**Figure 4:**
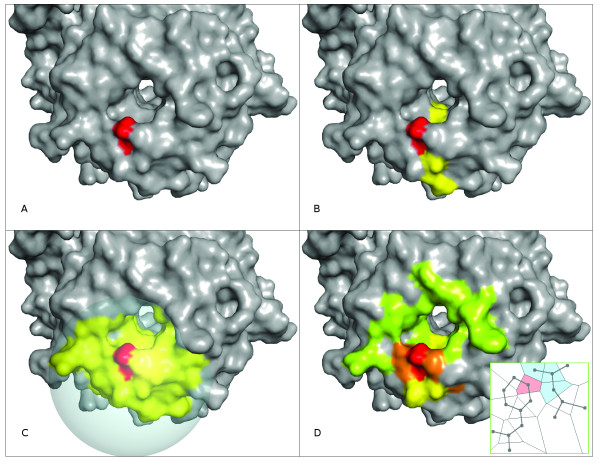
**Different definitions of residues' structural environment or neighbourhood**. (A) Single residue (red), i.e. no environment. (B) 9-residue sliding window (as in Sikic et al. [25]); central residue is shown in red and flanking residues in yellow. (C) Euclidean distance cut-off; residues enclosed in a sphere of radius R = 15 Angstroms (yellow) as in Porollo et al. [20], centred on the given residue (red). (D) Voronoi Diagrams; residue of interest (red) with colour gradient showing neighbouring residues; orange: residues sharing more than 16 edges with residue of interest; yellow: between 8 to 16; green: less than 8. Inset shows the 2D projection of a VD between two residues.

There are different strategies to determine neighbouring residues or the environment of exposed residues, including Euclidean distance cut-offs or sliding windows (Figure 4AB-C). In this report, the environment of residues is defined using VDs. The underlying principle is to consider neighbouring residues as those that are visible to the given residue, i.e. share a common edge in the VD (Figure 4D). Thus, in the context of VDs, a pair of residues are said to be in contact when at least one pair of heavy atoms of each residue have a facet in common, which clearly differs from the classical definition that implies atomic interactions (either bonded or non-bonded). VDs were calculated using the qvoroni application of the Qhull package [36].

Given the nature of VDs, some residues will have more contacts than others depending on their specific location. To take this into account, a contact strength term *c_ij _*(2) was defined as follows. Let *a_i _*be a given residue and *{a_j_/j = 1, ..., n} *its neighbours. For each neighbour *a_j_*, let *N_ij _*be the number shared edges or contact pairs between *a_i _*and *a_j_*, then:

(1) $$N_{i}=\overset{n}{\underset{j=\text{1}}{\sum }}N_{ij}$$

is the total number of contact pairs of *a_i _*with all its neighbours. The strength of the contact *c_ij _*between the amino acids *a_i _*and *a_j _*is defined as:

(2) $$c_{ij}=\frac{N_{ij}}{N_{i}}$$

Thus, the clear advantages of using VDs to define the environment are twofold: (i), thresholds are not required, as contacts are based on visibility between residues rather than Euclidian distances or sliding window; and (ii), a weighting factor (2) can be defined based on the number of contacts between residues.

### VORFFIP prediction algorithm

VORFFIP algorithm consists of two consecutive RF ensemble classifiers, named first-step and second-step RFs. In the first-step RF, residues and environment-based features are calculated and used as input variables. The scores yielded by the first-step RF are then decomposed into a number of new input variables that together with the previously calculated features are inputted to the second-step RF to calculate the final scores (see Figure 1 for an overview and schematic representation of the method). The randomForest package [37] implemented in R (http://www.r-project.org/) was used to train and compute decision trees.

#### First-step Random Forest

The input variables for the first-step RF include residue and environment-based information.

##### Residue-based features

A set of different attributes was used to characterize exposed residues. These are classified into four groups: (i) structure-based; (ii) energy-based; (iii) evolutionary-based; and (iv) experimental B-factors. A full description of each individual feature is given in additional file 1, Material and Methods section. For a given amino acid *a_i _*the set of features is defined as:

(3) $$F_{i}=\left\{f_{ik}∕k\in K\right\}$$

where *K *is an index set of all the features listed in additional file 1, Material and Methods section.

##### Environment-based features

The neighbouring residues were identified using VDs as described previously. Three metrics were devised to account for the environment: the EF vector (5), the Contact Description Vector (CDV) (6); and the Environment Description Matrix (EDM) (7).

The EF vector (5) is similar to the F vector (3) but relates to the neighbouring residues and is weighted by *c_ij _*(2). Given the residue *a_i _*and *{a_j/_j = 1, ..., n} *its neighbours, for each *k *feature of *k ∈ K*, a environment *ef_ik _*element [4] is defined as:

(4) $$ef_{ik}=\overset{n}{\underset{j=\text{1}}{\sum }}c_{ij}f_{jk}$$

where *f_jk _*is the value of the *k^th ^*feature for residue *a_j _*weighted by *c_ij _*(2). Then, EF vector is the set of the environment features for residue *a_i _*defined as:

(5) $$EF_{i}=\left\{ef_{ik}∕k\in K\right\}$$

The CDV (6) and EMD (7) metrics quantify the physicochemical properties of the environment, i.e. the different residue types that comprise the environment. CDV accounts for the residue types that are in contact with the residue under consideration and is a 20-tuple vector where each element represents a residue type weighted by *c_ij _*(2). Formally, let *a_i _*be the residue being considered and *{a_j/_j = 1, ..., n} *its neighbours, then the *l^th ^*element of CDV is:

(6) $$cdv_{l}=\underset{a_{j}\equiv type_{l}}{\sum }c_{ij}$$

where *a_j _*is a neighbouring residue of the type *type_l _*(i.e. Ala, Cys, etc).

As CDV does not contain information regarding contacts within residues in the environment (i.e. excluding the residue being predicted, e.g. *a_i _*as in CDV), EDM is used. Thus, EDM (7) is a 20 by 20 matrix where the component *edm_lk _*represents the normalised number of contacts between residues of type *type_l _*and *type_k _*resulting from any pair of residues in the environment. Formally, *edm_lk _*would be:

(7) $$edm_{lk}=\frac{\text{1}}{M_{i}}\underset{a_{r}}{\sum }\underset{a_{s}}{\sum }N_{rs}$$

where *M_i _*is:

(8) $$M_{i}=\overset{n}{\underset{r=\text{1}}{\sum }}\overset{n}{\underset{s>r}{\sum }}N_{rs}$$

and where *a_r _*is a residue of type *type_l _*and *a_s _*of type *type_k _*and *N_rs _*is the number of contacts between residues *a_r _*and *a_s_*.

#### Second-step Random Forest

As a result of the first-step RF, score are assigned to each residue. The second-step RF makes use of this information in the form of score values (*s_i_*), environmental scores (*es_i_*) (9), the contact score vector (CSV) (10), and maximum-minimum score (Mms) values (11) that are added to the variables listed above to output a final score (Figure 2).

Let *a_i _*be residue under consideration and *{a_j/_j = 1, ..., n} *its neighbours, then environmental score value *es_i _*of residue *a_i _*is defined as:

(9) $$es_{i}=\overset{n}{\underset{j=\text{1}}{\sum }}c_{ij}s_{j}$$

where *s_j _*is the score assigned by first-step RF to residue *a_j _*normalized by *c_ij _*(2).

The environmental scores can be decomposed into values amongst the different residue types, and thus *l^th ^*of CSV is defined as:

(10) $$csv_{l}=\underset{a_{j}\equiv type_{l}}{\sum }c_{ij}s_{j}$$

where *a_j _*is a neighbour of *a_i _*and *type_l _*corresponds to the *l^th ^*residue type. CSV is analogous to CDV described above, but accounts for contribution to the environmental score of the different residue types. Finally, Mms (11) or the Maximum- minimum scores is also included:

(11) $$Mms=\left\{\left(max\left\{s_{j}\right\},c_{ij}\right),\left(min\left\{s_{j}\right\},c_{ij}\right),\left(max\left\{s_{ij}p_{j}\right\},c_{ij}\right),\left(min\left\{s_{ij}p_{j}\right\},c_{ij}\right)\right\}$$

where *s_j _*is the score assigned by the first-step RF to the neighbouring residue *a_j_*.

### Assessing the performance of the method

Five widely used statistical measures were used to evaluate the performance of the method: Recall (R), Precision (P), the Matthew Correlation Coefficient (MCC), Q2 quartile, and the F1 score. The statistical analysis of ROC curves was performed using the StAR program [38]. Further information is given in additional file 1, Material and Methods section.

## Authors' contributions

JS: design, acquisition, analysis and interpretation of data, and writing of the manuscript. PFJ: supervision, analysis of data, and writing of the manuscript. NFF: concept, design, supervision, analysis and interpretation of data, and writing of the manuscript. All authors read and approved the final manuscript.

## Supplementary Material

Additional file 1 **Supplementary data**. This file includes additional information regarding methods and databases and extra tables and figures in portable document format (pdf). Click here for file
