# Supplementary material for: Improving the prediction of protein binding sites by combining heterogeneous data and Voronoi diagrams
Source: BMC Bioinformatics. 2011 Aug 23;12:352. doi: 10.1186/1471-2105-12-352 (PMC3171731; doi:10.1186/1471-2105-12-352)
Supplement: Additional file 1 — Supplementary data. This file includes additional information regarding methods and databases and extra tables and figures in portable document format (pdf). [file 1471-2105-12-352-S1.PDF]

SUPPLEMENTARY MATERIAL

"Improving the prediction of protein binding sites by combining heterogeneous data and Voronoi Diagrams" by Segura et al.

Material and Methods

Datasets and definitions of protein interfaces

Five datasets of protein complexes, termed O333, S435, S149, W025 and B100, were used for benchmarking and comparison purposes. Different definitions of protein interfaces were used depending on the specific dataset to accommodate previous authors using different definitions to locate protein interfaces; some interfaces have been defined by a Euclidean distance to different protein chains, others have defined interface residues as those forming atomic interactions with other proteins in the complex. As VORFFIP is being compared to these methods, the same methodology was used for consistency and comparability of the results.

The O333 dataset corresponds that compiled by Ofran et al. (Ofran and Rost, 2003) and used by Sikic et al. (Sikic, et al., 2009). Residues in dataset O333 were considered to be part of a protein interface if closer than 6 Å to any heavy atom in a neighbouring non-homologous chain. The datasets S435 and S149 correspond to the two sets derived by Porollo et al., used to train and test SPPIDER (Porollo and Meller, 2007). The interfaces are defined in terms of the relative surface area (RSA) and accessibility surface area (ASA) changes between the unbound and bound structures. Any residue whose RSA changes more than 4% between unbound and the bound complex and has a ASA larger than 5 $Å^2$, was considered to be part of a protein interface. The dataset W025 corresponds to both Benchmark 1.0 (Chen, et al., 2003) and 2.0 (Mintseris, et al., 2005) sets used to benchmark WHISCY (de Vries, et al., 2006). The interface residues were defined using DIMPLOT(Wallace, et al., 1995) with default parameters. Dataset B100 corresponds to Benchmark 3.0 (Hwang, et al., 2008) after discarding antigen antibody complexes and was used as an independent set to benchmark VORFFIP under different conditions, i.e. input data and environment definitions. Interfaces were located using DIMPLOT (Wallace, et al., 1995) with default parameters.

Voronoi Diagrams

The formal definition of Voronoi Diagrams (VDs) is as follows: given a finite set of points *S* in the 3D-space, the VD is a spatial partition defined by those regions where points are closer to a particular element of *S* than to any other. In other words, the VD splits the space into cells where: (i) inside each cell there is only one element of *S,* and (ii) all the points inside a cell are closer to this particular element of *S* than to any other element of *S*. Therefore, considering the set of cells of the VD, two cells will be neighbours if their borders share a common edge. Translating this concept into proteins, each cell of the VD will contain an individual atom (hydrogen atoms not considered) and two atoms will be neighbours if their respective cells are neighbours in the VD. This property can be extended to the residue level: two given residues *i* and *j* would be neighbours if any of their atoms are neighbours, i.e. share a common edge in the Voronoi Diagram partition. Note that only exposed residues (i.e. positive accessible surface area as defined by DSSP(Kabsch and Sander, 1983)) are considered.

VORFFIP algorithm

Random Forest classifier

Random Forests (RFs) (Breiman, 1984) is an ensemble classifier that consists of several decision trees. As with other ensemble classifiers, a RF is a machine-learning algorithm that requires a training set to learn the different parameters that will build each individual decision tree. An independent dataset (control set) is then used to test the accuracy of the method. From an initial training set the decision trees are constructed as follows. First, a bootstrap sample is produced from the original training set. Secondly, at each node, *m* variables are selected at random and the best split by CART algorithm (Breiman, 1984). Finally, the tree is grown to the largest possible extent. The randomForest package(Liaw and Wiener, 2002) implemented in R (http://www.r-project.org/) was used to train and compute decision trees. Further technical details includes: a number of 200 decision trees was created for each forest and the number of random variables selected in each node for tree construction was set to

√(n), where n was the total number of random variables of the forest. This value ensured acceptable prediction strength of individual trees of the forest and a low correlation value between them.

Residue-based features

Structure-based

Structure-based features include the residue type, secondary structures as defined by DSSP(Kabsch and Sander, 1983) and a set of structural attributes as defined by PSAIA(Mihel, et al., 2008) that includes:

- Hydrophobicity
- ASA (accessible surface area)
- Relative ASA
- Backbone ASA
- Relative backbone ASA
- Side chain ASA
- Relative side chain ASA
- Non-polar ASA
- Relative non-polar ASA
- Polar ASA
- Relative polar ASA
- DPX (depth index)
- Maximal DPX
- CX (protrusion index)
- Minimal CX
- Maximal CX

Energy-based

FoldX(Guerois, et al., 2002) was used to compute the different residue-based energy terms that include:

- Polar solvent exposure energy
- Hydrophobic solvent exposure energy
- Van der Waals energy
- Side chain entropy
- Backbone entropy
- Side chain occupancy

- Backbone occupancy
- Side chain hydrogen bond energy
- Backbone hydrogen bond energy
- Electrostatic energy

Evolutionary-based

Evolutionary-based features include residue conservation index as calculated by AL2CO (Pei and Grishin, 2001) and the regional conservation index as defined by Landgraf et al. (Landgraf, et al., 2001).

Crystallographic B-factors

A B-factor *Z-score* [1] was calculated as described Yuan et al. (Yuan, et al., 2003):

$$NB_r = \frac{B_{r\alpha} - \mu(B)}{\sigma(B)} \quad [1]$$

where $B_{r\alpha}$ the B-factor value of the alpha carbon alpha, $\mu(B)$ is the B-factor average over the protein chain and $\sigma(B)$ the standard deviation.

Assessing performance

Five widely used statistical measures were used to assess the performance of the method. These were: Recall [2], Precision [3], the Matthews Correlation Coefficient (MCC)[4], the second quartile (Q2)[5], and the F1 score [6]. Formally,

$$Recall = \frac{TP}{TP + FN} \quad [2]$$

$$Precision = \frac{TP}{TP + FP} \quad [3]$$

$$MCC = \frac{TP \times TN - FP \times FN}{\sqrt{(TP + FN)(TP + FP)(TN + FP)(TN + FN)}} \quad [4]$$

$$Q2 = \frac{TP + TN}{TP + FP + TN + FN} \quad [5]$$

$$F1 = \frac{2TP}{2TP + FN + FP} \quad [6]$$

where TP is the number of true positives, TN true negatives, FP false positives, FN the number of false negatives.

ROC curves were analyzed using the program StART(Vergara, et al., 2008) to assess the statistical significances of the differences between AUC values obtained using different combinations of features and environment definitions. StART program relies on a non-parametric test that accounts for the correlation of the ROC curves.

RESULTS

Table S1. Statistical analysis of ROC curves using StAR(Vergara, et al., 2008). The header and first columns column indicates the combination of features used to compute the ROC curves: structural (s), energy terms (e), conservation (c), and B-factors (b). In this test, Voronoi Diagram environment was used. The lower diagonal contains the *p-values* that represent the statistical significance of the difference between AUC.

	**s**	**s+e**	**s+e+c**	**s+e+c+b**
**s**	N.A.			
**s+e**	0,06	N.A.		
**s+e+c**	3,66E-022	1,50E-028	N.A.	
**s+e+c+b**	2,76E-023	1,27E-029	0,58	N.A.

Table S2. Statistical analysis of ROC curves using StAR(Vergara, et al., 2008). The header and first columns column indicates the environment used to compute the ROC. In this test all residue features (structural, energy terms, conservation, and B-factors) were used. The lower diagonal contains the *p-values* that represent the statistical significance of the difference between AUC.

	**voronoi**	**window**	**distance**	**no enviro.**
**voronoi**	N.A.			
**window**	6,60E-009	N.A.		
**distance**	1,04E-035	5,27E-012	N.A.	
**no enviro.**	4,80E-086	7,48E-068	3,11E-017	N.A.

Table S3. Matthew Correlation coefficient, F1 score, Precision, and Recall values for different combinations of features and environment definitions. The test consisted of a 5-fold cross validation using dataset B100 where interface residues were defined using DIMPLOT (Wallace, et al., 1995). The first column indicates the combination of features used: structural (s), energy terms (e), conservation (c), and B-factors (b). The table also shows the MCC (a), F1 score (b), Precision (c), and Recall (d) values for the different type of environment descriptors: VD, sphere (15 Å cut-off), sliding window (9 residues), and single residues (no environment).

	**Voronoi Diagrams**				**Sphere**				**Sliding Window**				**Single**			
	MCC[a]	F1[b]	P[c]	R[d]	MCC[a]	F1[b]	P[c]	R[d]	MCC[a]	F1[b]	P[c]	R[d]	MCC[a]	F1[b]	P[c]	R[d]
**s**	0.27	0.33	0.28	0.41	0.24	0.12	0.92	0.07	0.27	0.25	0.56	0.16	0.21	0.13	0.66	0.07
**e**	0.24	0.21	0.56	0.13	0.26	0.16	0.85	0.09	0.31	0.23	0.79	0.14	0.22	0.14	0.73	0.08
**c**	0.23	0.29	0.28	0.31	0.26	0.15	0.91	0.08	0.33	0.26	0.82	0.15	0.16	0.11	0.49	0.06
**b**	0.25	0.16	0.75	0.09	0.26	0.14	0.94	0.08	0.34	0.25	0.86	0.15	0.09	0.15	0.16	0.15
**s+e**	0.26	0.32	0.27	0.39	0.24	0.13	0.91	0.07	0.31	0.29	0.55	0.21	0.21	0.1	0.95	0.05
**s+c**	0.31	0.36	0.37	0.36	0.26	0.31	0.33	0.29	0.29	0.32	0.44	0.25	0.26	0.29	0.39	0.23
**s+b**	0.27	0.33	0.27	0.43	0.24	0.13	0.89	0.07	0.27	0.23	0.6	0.14	0.21	0.09	0.97	0.05
**e+c**	0.29	0.33	0.38	0.29	0.27	0.18	0.81	0.11	0.32	0.31	0.5	0.22	0.24	0.16	0.76	0.09
**e+b**	0.25	0.29	0.34	0.26	0.25	0.14	0.88	0.08	0.32	0.24	0.79	0.14	0.22	0.12	0.83	0.07
**c+b**	0.24	0.29	0.32	0.27	0.27	0.18	0.84	0.1	0.31	0.23	0.77	0.13	0.19	0.09	0.87	0.05
**s+e+c**	0.31	0.36	0.37	0.35	0.26	0.31	0.36	0.27	0.33	0.32	0.46	0.25	0.26	0.31	0.29	0.34
**s+e+b**	0.27	0.33	0.27	0.44	0.23	0.12	0.92	0.06	0.26	0.23	0.59	0.14	0.21	0.14	0.7	0.07
**s+c+b**	0.31	0.36	0.35	0.46	0.27	0.31	0.38	0.26	0.31	0.34	0.41	0.29	0.26	0.31	0.3	0.33
**e+c+b**	0.29	0.35	0.33	0.36	0.26	0.22	0.57	0.14	0.32	0.31	0.49	0.22	0.25	0.17	0.72	0.11
**s+e+c+b**	0.31	0.35	0.47	0.31	0.26	0.31	0.34	0.29	0.31	0.33	0.45	0.26	0.26	0.31	0.33	0.28

Table S4. Matthew Correlation coefficient, F1 score, Precision, and Recall values for different distance cut-offs and Voronoi Diagrams (VDs). The test consisted on a 5-fold cross validation using dataset B100 where interface residues were defined using DIMPLOT (Wallace, et al., 1995). The first column indicates the different distance cut-offs. The table also shows the AUC (a), MCC (b), F1 score (c), Precision (d), and Recall (e) values.

	**AUC[a]**	**MCC[b]**	**F1[c]**	**P[d]**	**R[e]**
**5**	0.81	0.24	0.29	0.31	0.19
**10**	0.82	0.28	0.33	0.32	0.23
**15**	0.82	0.26	0.31	0.34	0.21
**20**	0.79	0.22	0.25	0.36	0.14
**VDs**	0.85	0.31	0.35	0.47	0.31

Table S5. Result for individual proteins in W025 dataset. First column is the PDB code; second column chain ID and in brackets (number of interface residues/number of surface residues); third, fourth and fifth predictions for VORFFIP, WHISCY and WHISMATE, respectively (predicted number of true positives residues/total predicted residues); sixth, seventh and eighth column precision (P) and recall (R) values for each method

					**VORFFIP**		**WHISCY**		**WHISMATE**	
		**VORFFIP**	**WHISCY**	**WHISMATE**	R	P	R	P	R	P
1ACB	E (13/162)	3/12	6/16	7/17	0.23	0.25	0.46	0.38	0.54	0.41
	I (10/61)	7/24	5/7	8/15	0.7	0.29	0.5	0.71	0.8	0.53
1AVX	A (18/143)	2/2	5/6	7/13	0.11	1	0.28	0.83	0.39	0.54
	B (9/124)	8/19	0/2	0/5	0.88	0.42	0	0	0	0
1AY7	A (10/78)	10/29	0/2	2/11	1	0.34	0	0	0.2	0.18
	B (9/68)	9/10	6/7	7/8	1	0.9	0.67	0.86	0.78	0.88
1BVN	P (19/276)	6/7	6/17	3/10	0.31	0.85	0.32	0.35	0.16	0.3
	T (14/62)	10/10	4/4	5/8	0.71	1	0.29	1	0.36	0.63
1CGI	E (22/161)	6/13	7/15	9/15	0.27	0.46	0.32	0.47	0.41	0.6
	I (11/43)	10/12	1/6	4/11	0.9	0.83	0.09	0.17	0.36	0.36
1D6R	A (12/145)	3/10	4/11	6/18	0.25	0.3	0.33	0.36	0.5	0.33
	I (8/53)	3/18	0/1	2/31	0.37	0.16	0	0	0.25	0.06
1DFJ	I (15/274)	4/12	3/9	3/5	0.26	0.33	0.2	0.33	0.2	0.6
	E (16/98)	11/25	3/12	3/12	0.68	0.44	0.19	0.25	0.19	0.25
1E6E	A (19/302)	8/20	13/52	4/15	0.42	0.4	0.68	0.25	0.21	0.27
	B (19/79)	10/19	11/20	11/22	0.52	0.52	0.58	0.55	0.58	0.5
1EAW	A (15/149)	0/4	7/15	8/21	0	0	0.47	0.47	0.53	0.38
	B (7/48)	7/17	1/1	2/2	1	0.41	0.14	1	0.29	1
1EWY	A (6/197)	5/14	5/28	2/7	0.83	0.35	0.83	0.18	0.33	0.29
	C (7/73)	2/8	4/12	4/12	0.28	0.25	0.57	0.33	0.57	0.33
1EZU	A (15/215)	8/13	8/19	1/8	0.53	0.61	0.53	0.42	0.07	0.13
	C (23/144)	9/19	6/11	6/20	0.39	0.47	0.26	0.55	0.26	0.3
1F34	A (20/204)	3/13	4/10	4/5	0.15	0.23	0.2	0.4	0.2	0.8
	B (21/103)	2/16	0/0	0/1	0.09	0.12	0	0	0	0
1HIA	A (15/140)	7/21	3/3	4/8	0.46	0.33	0.2	1	0.27	0.5
	I (10/46)	10/32	1/2	5/18	1	0.31	0.1	0.5	0.5	0.28
1MAH	A (12/296)	0/15	1/11	1/5	0	0	0.08	0.09	0.08	0.2
	F (12/56)	7/13	0/3	0/4	0.58	0.53	0	0	0	0
1PPE	E (15/146)	10/22	5/11	7/17	0.66	0.45	0.33	0.45	0.47	0.41
	C (9/29)	9/20	1/1	5/19	1	0.45	0.11	1	0.56	0.26
1TMQ	A (19/273)	4/10	4/12	1/4	0.21	0.4	0.21	0.33	0.05	0.25
	B (14/87)	7/8	2/5	3/6	0.5	0.87	0.14	0.4	0.21	0.5
1UDI	E (12/147)	12/18	4/10	2/7	1	0.66	0.33	0.4	0.17	0.29
	I (13/66)	7/13	0/0	2/6	0.53	0.53	0	0	0.15	0.33
1WQ1	G (13/213)	0/11	5/6	0/0	0	0	0.38	0.83	0	0

	R (14/113)	14/26	9/22	7/16	1	0.53	0.64	0.41	0.5	0.44
2MTA	L (11/280)	0/19	2/4	2/3	0	0	0.18	0.5	0.18	0.67
	A (9/77)	6/7	3/5	4/7	0.66	0.85	0.33	0.6	0.44	0.57
2PCC	A (4/192)	2/13	0/5	0/7	0.5	0.15	0	0	0	0
	B (6/81)	5/16	0/10	0/14	0.83	0.31	0	0	0	0
2SIC	E (17/161)	8/9	6/9	4/5	0.47	0.88	0.35	0.67	0.24	0.8
	I (12/85)	5/14	0/10	2/14	0.41	0.35	0	0	0.17	0.14
2SNI	E (15/162)	6/10	7/11	5/6	0.4	0.6	0.47	0.64	0.33	0.83
	I (6/53)	6/17	2/3	2/4	1	0.35	0.33	0.67	0.33	0.5
7CEI	B (10/98)	4/12	0/4	0/2	0.4	0.33	0	0	0	0
	A (12/71)	8/11	1/3	1/4	0.66	0.72	0.08	0.33	0.08	0.25
1AVW	A (19/144)	8/14	8/10	9/14	0.42	0.57	0.42	0.8	0.47	0.64
	B (11/119)	8/15	0/3	1/9	0.72	0.53	0	0	0.09	0.11
1BRC	E (14/140)	3/8	6/10	7/14	0.21	0.37	0.43	0.6	0.5	0.5
	I (8/49)	8/13	2/3	4/5	1	0.61	0.25	0.67	0.5	0.8

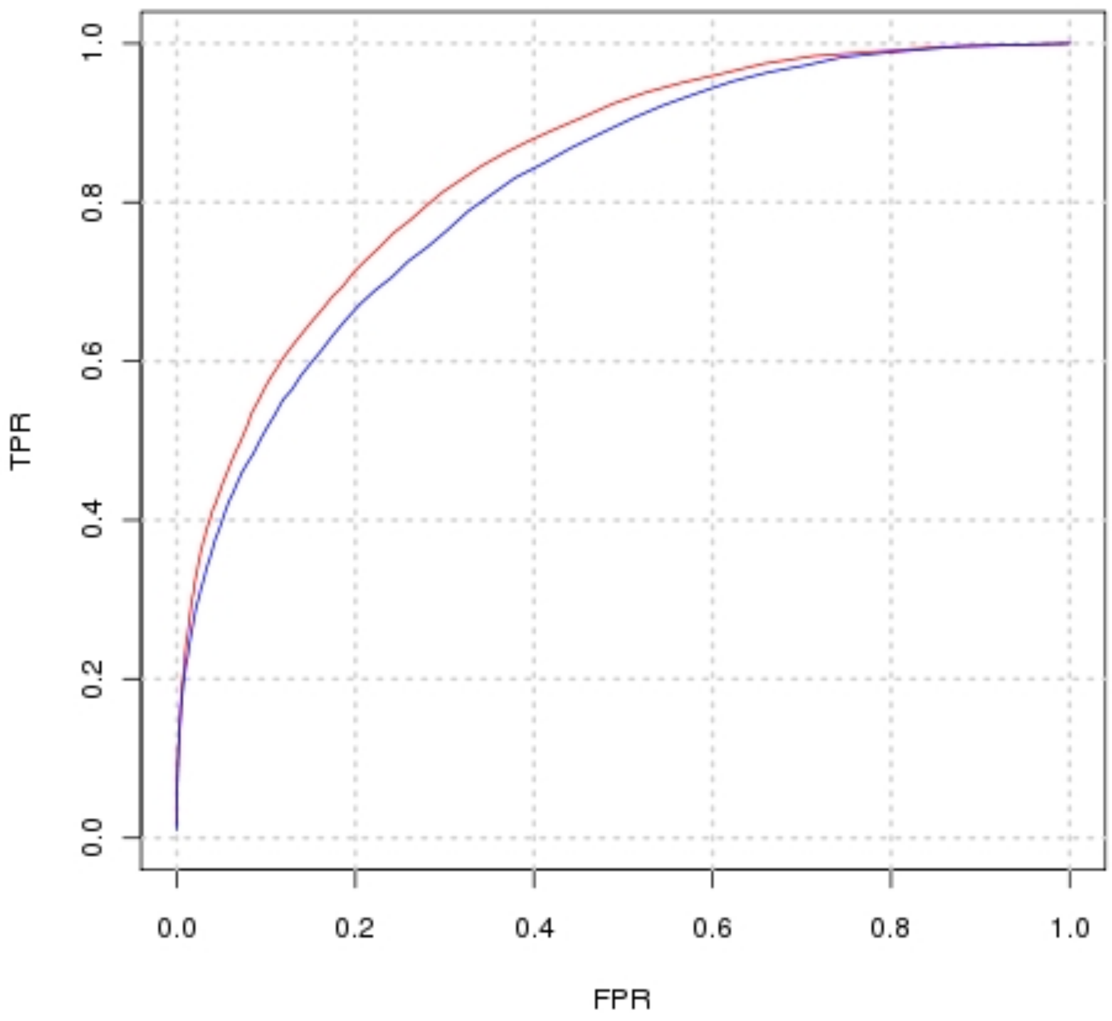

Figure S1. ROC curves for the first-step (blue) and second-step (red) RF in a 5-fold cross validation benchmark using B100 dataset. X-axis and Y-axis represent false positive and true positive rates, respectively.

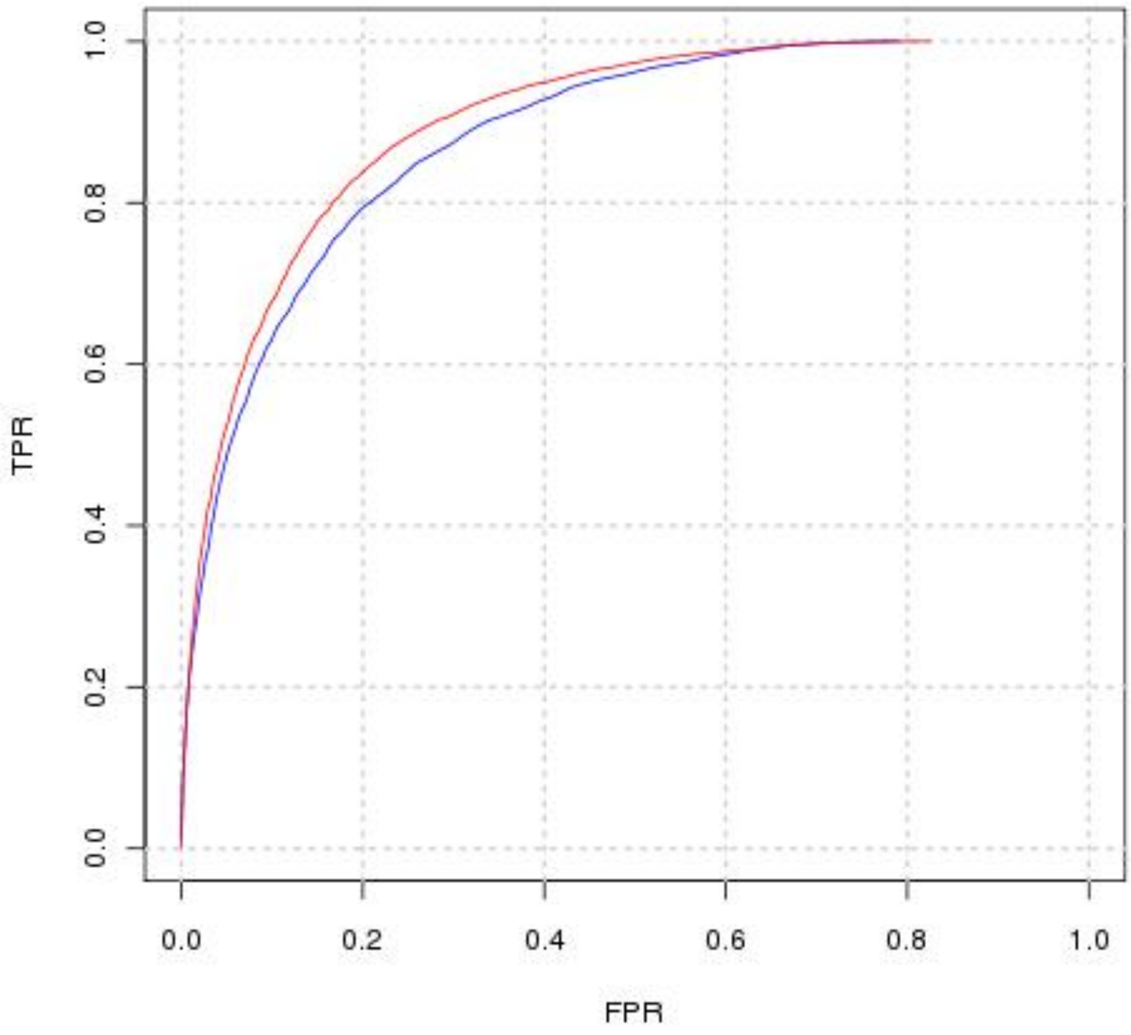

Figure S2. ROC curves using SPPIDER datasets (Porollo and Meller, 2007). X-axis and Y-axis represent false positive and true positive rates, respectively. The method was trained using S435 data set and tested with dataset S149; interface residues were defined in terms of the relative surface area (RSA) and accessibility surface area (ASA) changes between the unbound and bound structures as described in the original report (Porollo and Meller, 2007). Blue and red curves are the result for the first-step and second-step RF, respectively. ROC curves obtained using SPPIDER are available in the original publication (Porollo and Meller, 2007).

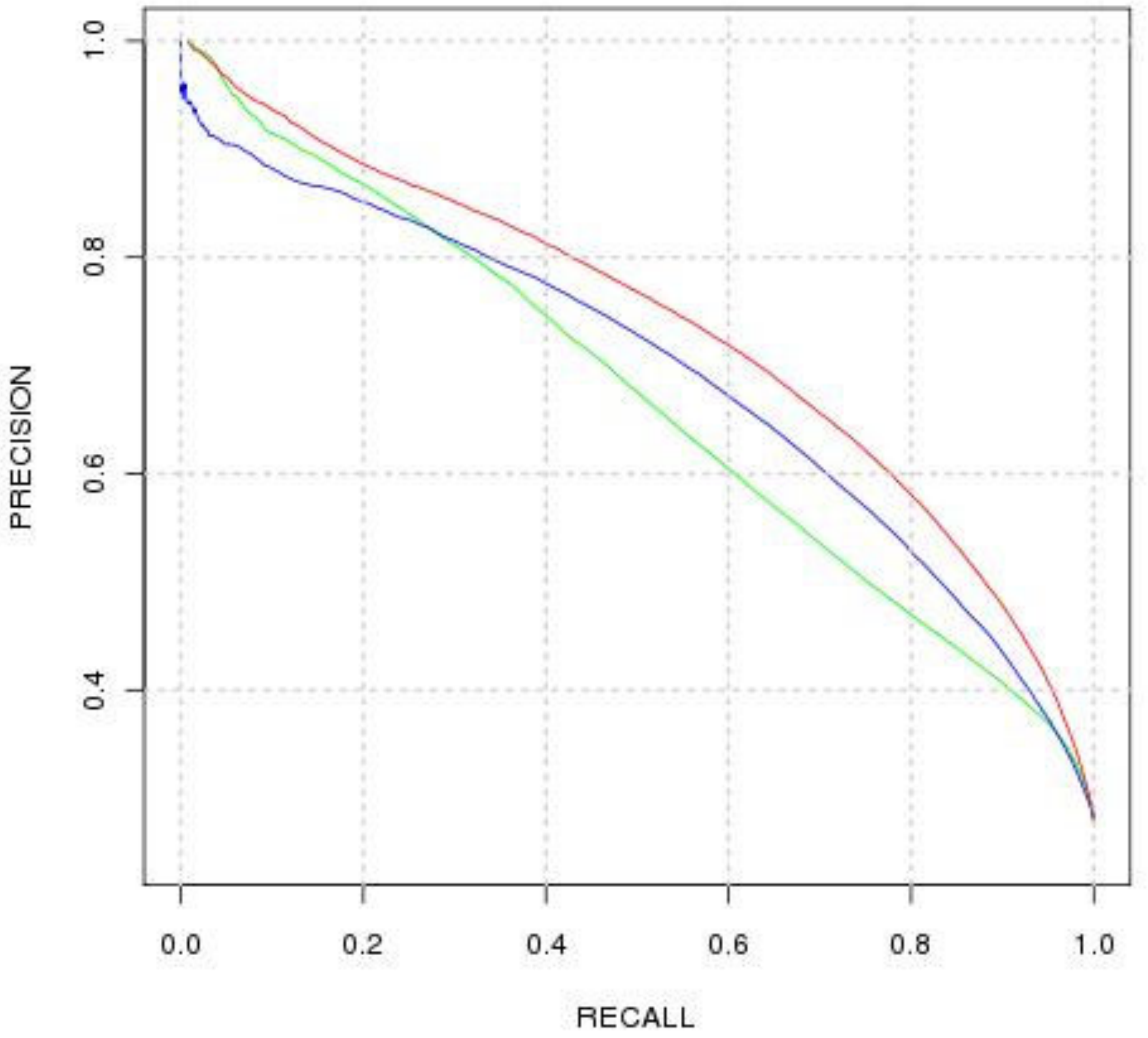

Figure S3. Precision versus recall curve on the O333 dataset following same benchmark procedure described by Sikić et al.(Sikic, et al., 2009). X-axis and Y-axis represent recall and precision respectively. Solid green, blue and red lines represent Sikić et al.(Sikic, et al., 2009), first-step, and second-step RF, respectively.

REFERENCES

Breiman, L. (1984) *Classification and Regression Trees*. CHAPMAN & HALL/CRC.

Chen, R.*, et al.* (2003) A protein-protein docking benchmark, *Proteins*, **52**, 88-91.

de Vries, S.J., van Dijk, A.D.J. and Bonvin, A.M.J.J. (2006) WHISCY: what information does surface conservation yield? Application to data-driven docking, *Proteins*, **63**, 479-489.

Guerois, R., Nielsen, J.E. and Serrano, L. (2002) Predicting changes in the stability of proteins and protein complexes: a study of more than 1000 mutations, *J Mol Biol*, **320**, 369-387.

Hwang, H.*, et al.* (2008) Protein-protein docking benchmark version 3.0, *Proteins*, **73**, 705-709.

Kabsch, W. and Sander, C. (1983) Dictionary of protein secondary structure: pattern recognition of hydrogen-bonded and geometrical features, *Biopolymers*, **22**, 2577-2637.

Landgraf, R., Xenarios, I. and Eisenberg, D. (2001) Three-dimensional cluster analysis identifies interfaces and functional residue clusters in proteins, *J.Mol.Biol.*, **307**, 1487.

Liaw, A. and Wiener, M. (2002) Classification and Regression by randomForest, *R News*, **2**, 18-22.

Mihel, J.*, et al.* (2008) PSAIA - protein structure and interaction analyzer, *BMC Struct Biol*, **8**, 21.

Mintseris, J.*, et al.* (2005) Protein-Protein Docking Benchmark 2.0: an update, *Proteins*, **60**, 214-216.

Ofran, Y. and Rost, B. (2003) Analysing six types of protein-protein interfaces, *J Mol Biol*, **325**, 377-387.

Pei, J. and Grishin, N.V. (2001) AL2CO: calculation of positional conservation in a protein sequence alignment, *Bioinformatics*, **17**, 700-712.

Porollo, A. and Meller, J.Ç. (2007) Prediction-based fingerprints of protein-protein interactions, *Proteins*, **66**, 630-645.

Sikic, M., Tomic, S. and Vlahovicek, K. (2009) Prediction of protein-protein interaction sites in sequences and 3D structures by random forests, *PLoS Comput Biol*, **5**, e1000278.

Vergara, I.A.*, et al.* (2008) StAR: a simple tool for the statistical comparison of ROC curves, *BMC Bioinformatics*, **9**, 265.

Wallace, A.C., Laskowski, R.A. and Thornton, J.M. (1995) LIGPLOT: a program to generate schematic diagrams of protein-ligand interactions, *Protein Eng*, **8**, 127.

Yuan, Z., Zhao, J. and Wang, Z.-X. (2003) Flexibility analysis of enzyme active sites by crystallographic temperature factors, *Protein Eng*, **16**, 109-114.
